# Social participation and acceptance of disability in young and middle-aged breast cancer patients after surgery: A 6-month follow-up study

**DOI:** 10.1016/j.apjon.2023.100266

**Published:** 2023-07-04

**Authors:** Mengyao Zhu, Yiheng Zhang, Haiyan He, Lili Chen, Juanjuan Chen, Meifen Zhang

**Affiliations:** a School of Nursing, Sun Yat-sen University, Guangzhou, China; b Sun Yat-sen Memorial Hospital, Sun Yat-sen University, Guangzhou, China

**Keywords:** Young and middle-aged, Breast neoplasms, Social participation, Acceptance of disability, Longitudinal studies

## Abstract

**Objective:**

To describe the social participation and acceptance of disability (AOD) in young and middle-aged patients with breast cancer after surgery and their dynamic trajectories and to explore the critical factors associated with social participation.

**Methods:**

212 young and middle-aged patients with breast cancer after surgery were recruited for a 6-month follow-up study, and 158 of whom completed four surveys. Participants were asked to complete questionnaires including a general information questionnaire, Social Dysfunction Screening Scale, and Adaptation of Disability Scale Revised at baseline, and at 1, 3, and 6 months. T-test and chi-square test were used to analyze the difference in baseline data. Linear generalized estimating equations were used to analyze the dynamic trend and influencing factors. The Cochran-Armitage trend test was used to analyze the trend of the incidence of social function defects.

**Results:**

The status of social participation in patients after breast cancer surgery was poor, and 77.9%, 59.3%, 45.9%, and 29.1% had social function defects, respectively. The AOD was at a moderate level. Both social participation and AOD showed a trend of dynamic improvement. Age (*P* ​= ​0.044), residence (*P* ​= ​0.007), surgery type (*P* ​= ​0.043), postoperative chemotherapy (*P* ​= ​0.003), and AOD (*P* < 0.001) were the key factors associated with social participation.

**Conclusions:**

Medical staff should focus on elderly patients, who lived outside the provincial capital city, received total mastectomy, or modified radical mastectomy and postoperative chemotherapy. AOD might be an important potential avenue for improving the social participation level of young and middle-aged patients with breast cancer after surgery.

## Introduction

Breast cancer, the most common malignant tumor threatening women's health, shows an increasing tendency to affect younger people, including a high proportion of young and middle-aged individuals.^1^ In recent years, most patients with breast cancer have been able to achieve early diagnosis, early treatment, and long-term survival. In long-term survival process after surgery, how to best maintain a healthy psychological state and provide maximal support to patients with breast cancer in returning to society has become the focus among the nursing staff. Previous studies^2^ have shown that the first 6 months after surgery is a critical period for patients with breast cancer to adapt to psychosocial conditions. Therefore, the present study also focuses on investigating patients' psychological status and social behavior within half a year after surgery.

The ultimate goal of rehabilitation is to enable patients to recover and maintain functional independence, so as to achieve the greatest possible participation, and finally return to normal life and society. Social participation refers to the subjective experiences and behavioral processes in which individuals actively participate in family and social activities and integrate into the social environment, thus objectively reflecting patients’ physical and mental conditions.^3^ However, the psychosocial problems caused by the diagnosis and treatment of young and middle-aged patients with breast cancer are more prominent, and their social participation after surgery is poor, and 70% of patients have social function defects after surgery.^4^^,^^5^ Patients usually show a decline in their interest in the external matters and a sense of responsibility for family and work, a lack of planning for the future, their unwillingness to participate in social activities, and so on,^4^^,^^5^ thereby affecting their physical and mental recovery and quality of life,^6^^,^^7^ hindering their return to normal life and work, increasing the burden of family care and economic burden,^8^ and negatively affecting individuals, families, and society.^9^, ^10^, ^11^.

The degree of an individual's acceptance of their disability status is called acceptance of disability (AOD),^12^ which directly affects the psychosocial behavior and social interaction of patients toward disease.^13^ Individuals with a high level of AOD are often able to maintain a healthy and positive attitude toward life and have favorable social participation.^14^ However, as many as 90% of patients with breast cancer have moderate AOD after surgery.^13^, ^14^, ^15^ Increasing patients' AOD level can help them better cope with the physical and mental changes caused by the disease, thus promoting recovery and return to society.

The social participation of patients with cancer and AOD of disabled patients have attracted the attention of many researchers. However, research on patients with breast cancer remains in its infancy, and most studies have been cross-sectional. However, most patients with breast cancer are women, including a large group of young and middle-aged patients, and the psychosocial problems caused by diagnosis and treatment are prominent^4^ and warrant research focus. The course of the disease is a predictor of social participation and AOD of disabled patients,^16^, ^17^, ^18^, ^19^ but no relevant study has been conducted in patients with breast cancer.

To better understand the psychosocial adaptation of patients in different critical periods after surgery and provide a theoretical basis to help patients with breast cancer to better integrate into society, in the current study, we assessed the following aspects in young and middle-aged patients with breast cancer within 6 months after surgery:

(1) Dynamic trends in social participation, AOD, and their correlation;
(2) Dynamic influences of AOD on social participation;
(3) Critical factors influencing social participation.

## Methods

### Study design

From July 2021 to July 2022, we conducted a 6-month follow-up survey of 212 patients with breast cancer and treated at a tertiary hospital in Guangdong Province, China. Participants completed questionnaires at baseline (from operation to discharge, T0) and after 1 (T1), 3 (T2), and 6 (T3) months of follow-up. The social participation and AOD of patients were collected.

### Study participation

Patients included in this study were those who were (1) diagnosed with primary breast cancer and underwent surgery in our hospital within 1 week, (2) older than 18 years and younger than 59 years, and (3) aware of their own disease diagnosis.

The exclusion criteria were (1) past or present serious disease, (2) with recurrence, metastasis or other malignant tumors, and (3) participation in other studies.

### Sample size

According to G∗Power 3.1 software, a single group of repeated measurement commands was selected, *α* ​= ​0.05, 1-*β* ​= ​0.9, *f* ​= ​0.14,^20^ number of measurements ​= ​4, number of groups ​= ​1, and calculated *N* ​= ​92. Assuming a loss rate of 30% of samples in longitudinal study, we determined that a sample size of 132 patients was required.

### Measurements

#### Social function deficiency screening form

Social participation was measured by Social Function Deficiency Screening Form (SDSS), a 10-item multiple-choice self-report inventory.^21^ Response options ranged from 0 (not at all) to 2 (serious defect). Total scores ranged from 0 to 20. Scores ≥ 2 indicated social function defects and higher scores indicated more serious social function defects. Liu has applied SDSS in patients with breast cancer and reported a Cronbach's *α* value of 0.87.^22^ The Cronbach's *α* value in this study was 0.72.

#### Adaptation of disability scale revised

AOD was measured by Adaptation of Disability Scale Revised, a 32-item measure that designed to assess four dimensions: enlargement of scope of values (E), transformation from comparative values to asset values (T), containment of disability effect (C), and subordination of physique (S).^23^ Response options range from 1 (strongly disagree) to 4 (strongly agree). Total scores range from 32 to 128, with higher scores indicating higher acceptance. A low acceptance level was 32–64, a moderate level was 65–96, and a high level was 97–128. Chen has translated the scale into Chinese and reported a Cronbach's *α* value of 0.83.^24^ The Cronbach's *α* value in this study was 0.78.

#### Demographic questionnaire

The demographic variables included age, body mass index, religion, education, marital status, number of children, residence, living alone, family monthly income per capita, and work status during diagnosis.

#### Disease and treatment questionnaire

The disease- and treatment-associated variables included diagnosis type, focus location, surgery type, breast reconstruction, axillary lymph node dissection, discharge with portable drainage device, neoadjuvant chemotherapy, postoperative chemotherapy, radiotherapy, targeted therapy, and endocrine therapy.

### Data collection procedure

Eligible patients were identified at a tertiary hospital in Guangdong through a review of their medical records. The purpose and importance of this study were explained to patients, and their informed consent was obtained. Participants completed the demographic questionnaire and scale before discharge. To ensure credibility and confirmability, patients were provided with a quiet private room and sufficient time to fill out the questionnaires. The disease and treatment questionnaire was completed by researchers through reviewing of the medical records. The follow-up data were investigated online at 1, 3, and 6 months after operation. Researchers carefully examined all collected data and promptly confirmed any omitted or unclear responses with patients to ensure completeness and accuracy. When more than 20% of the content of a questionnaire was missing, the questionnaire was excluded from analysis. The study was reviewed by the Ethics Committee of Sun Yat-sen University (IRB No. SYSUIRBDA202001008).

### Data analysis

*T*-test or chi-square test was used to compare the balance of baseline data. The Cochran-Armitage trend test was used to analyze the trend of the incidence of social function defects. Considering the correlation and the phenomenon of missing among repeated measurement data,^25^ the generalized estimation equation (GEE) was used to analyze the dynamic trend of social participation and AOD and the factors influencing social participation. To consider as many potential influencing factors as possible, we included variables with *P* ​< ​0.100 in the univariate GEE in the multivariate GEE analysis. The working correlation structures with the minimum Quasi-likelihood under the independence model criterion (QIC) value were selected. When the QIC values of multiple matrices were equal, we selected the most stable unstructured matric. SPSS 25 was used for statistical analysis of the data, and difference was statistically significant (*P* ​< ​0.050).

## Results

### Baseline

A total of 215 questionnaires were distributed, and 212 valid questionnaires were returned (g-power ​= ​98.6%). A total of 189, 170, and 158 valid questionnaires were collected after 1, 3, and 6 months, respectively, thus resulting in a final follow-up rate of 74.5%. The reasons for loss to follow-up were two patients had recurrence and metastasis, 10 patients refused to participate, and 42 patients could not be contacted.

Ultimately, 158 participants completed all follow-up, which was considered as follow-up group, and the remaining participants were considered as lost group (*n* ​= ​54). The *t*-test or chi-square test was used to compare the baseline data for the two groups. Social participation and AOD showed no statistical difference at the baseline. In the demographic data, statistical differences were observed in religion (*P* ​= ​0.007), work status at diagnosis (*P* ​< ​0.001), and discharge with portable drainage device (*P* ​= ​0.018), whereas no statistical differences in other variables were identified.

### Participants’ characteristics

A total of 212 patients with breast cancer were enrolled in this study, with an average age of 45.44 years (SD ​= ​8.55). 59.4% of the patients had normal body mass index, 84.9% were married, 85.8% lived in cities and towns, and 52.8% were unemployed or retired at the time of diagnosis.

A total of 77.8% of the patients were diagnosed with invasive carcinoma, and 64.2% of the lesions were located on the left side. More than half of the patients received breast conserving surgery (54.2%), only 13.7% received breast reconstruction surgery, 32.1% underwent axillary lymph node dissection, 59.9% were given portable drainage devices when discharged, and 30.2% received preoperative neoadjuvant chemotherapy. A total of 54.7%, 52.4%, 50.9%, and 30.2% received postoperative chemotherapy, radiotherapy, endocrine therapy and targeted therapy, respectively. These data are shown in Table 1.

**Table 1:** Participants’ characteristics at baseline (*N* ​= ​212).

Variable	Mean ± SD	*n* (%)	Variable	*n* (%)
Age (years)	45.44 ​± ​8.55		Residence	
25∼		12 (5.7)	Provincial capital	67 (31.6)
31∼		50 (23.6)	Prefecture level city	58 (27.4)
41∼		83 (39.2)	County town	42 (19.8)
51∼59		67 (31.6)	Township	15 (7.1)
BMI (kg/m^2^)			Countryside	30 (14.2)
< 18.5		16 (7.5)	Whether to live alone	
18.5–24.0		126 (59.4)	Yes	9 (4.2)
≥ 24.0		70 (33.0)	Family monthly income per capita (¥)	
Religion			< 3000	53 (25.0)
Yes		12 (5.7)	3001–6000	81 (38.2)
No		200 (94.3)	≥ 6000	78 (36.8)
Number of children			Education level	
0		15 (7.1)	Junior high school or below	74 (34.9)
1		86 (40.6)	High school or technical secondary school	46 (21.7)
2		84 (39.6)	Junior college	36 (17.0)
3–5		27 (12.7)	Bachelor degree or above	56 (26.4)
Marital status			Work status during diagnosis	
Married		180 (84.9)	On job	100 (47.2)
Divorced		12 (5.7)	Unemployed or retired	112 (52.8)
Widowed		8 (3.8)	Discharge with portable drainage device	
Unmarried		12 (5.7)	Yes	127 (59.9)
Neoadjuvant chemotherapy			Breast reconstruction	
Yes		64 (30.2)	Yes	29 (13.7)
Postoperative chemotherapy			Axillary lymph node dissection	
Yes		116 (54.7)	Yes	68 (32.1)
Diagnosis type			Focus location	
Carcinoma *in situ*		47 (22.2)	Left side only	136 (64.2)
Invasive carcinoma		165 (77.8)	Right side only	69 (32.5)
Radiotherapy			Bilateral	7 (3.3)
Yes		111 (52.4)	Surgery type	115 (54.2)
Endocrine therapy			Breast preserving surgery	63 (29.7)
Yes		108 (50.9)	Modified radical mastectomy	34 (16.0)
Targeted therapy			Total mastectomy	
Yes		64 (30.2)		

### Social participation

#### Total SDSS scores

The total SDSS scores at T0, T1, T2, and T3 were 3.64 points (SD ​= ​2.68), 2.69 points (SD ​= ​2.59), 2.12 points (SD ​= ​2.59), and 1.27 points (SD ​= ​2.16), respectively. Moreover, 77.9%, 59.3%, 45.9%, 29.1% of the patients had social function defects, which decreased over time (*P* ​< ​0.001). The scores of social participation at two adjacent time points were statistically significant. These data are shown in Table 2 and Table 3.

**Table 2:** Longitudinal changes in the total score of social participation and AOD (Mean ± SD).

Variable	T0 (*N* ​= ​212)	T1 (*N* ​= ​189)	T2 (*N* ​= ​170)	T3 (*N* ​= ​158)	*P*
SDSS	3.64 ​± ​2.68	2.69 ​± ​2.59	2.12 ​± ​2.59	1.27 ​± ​2.16	< 0.001∗∗∗
				T3 < T2	< 0.001∗∗∗
				T2 < T1	0.015∗
				T1 < T0	< 0.001∗∗∗
ADS	88.70 ​± ​12.43	89.17 ​± ​15.45	91.22 ​± ​14.50	92.91 ​± ​12.64	0.004∗∗
				T3 > T2	0.032∗
				T2 > T1	0.157
				T1 > T0	0.802
ADS-E	26.82 ​± ​3.59	26.43 ​± ​4.78	26.24 ​± ​5.42	26.72 ​± ​4.01	0.432
ADS-C	24.92 ​± ​4.53	25.11 ​± ​5.62	25.98 ​± ​5.90	26.49 ​± ​4.88	0.003∗∗
ADS-T	25.75 ​± ​3.85	25.60 ​± ​5.36	26.45 ​± ​5.12	26.96 ​± ​4.35	0.015∗
ADS-S	11.21 ​± ​3.25	12.03 ​± ​2.89	12.55 ​± ​3.39	12.75 ​± ​2.99	< 0.001∗∗∗

∗*P* < 0.05; ∗∗*P* < 0.01; ∗∗∗*P* < 0.001; *P* value is the result of GEE and pairwise comparison.

ADS-E, enlargement of scope of values; ADS-C, containment of disability effect.

ADS-T, transformation from comparative values to asset values; ADS-S, subordination of physique; AOD, acceptance of disability; SDSS, Social Function Deficiency Screening Form.

**Table 3:** Longitudinal changes in functional defect rate [*n* (%)].

Variable	T0	T1	T2	T3	*P*
The total score of SDSS (≥ 2)	164 (77.9)	112 (59.3)	78 (45.9)	46 (29.1)	< 0.001∗∗∗
SDSS-work function (1–2)^a^	29 (29.0)	11 (19.6)	10 (19.2)	15 (20.5)	< 0.001∗∗∗
SDSS-marriage function (1–2)^b^	8 (4.4)	4 (2.5)	9 (5.7)	8 (5.4)	< 0.001∗∗∗
SDSS-parental function (1–2)^c^	11 (5.6)	11 (6.3)	22 (13.6)	16 (10.6)	< 0.001∗∗∗
SDSS-social withdrawal (1–2)	137 (74.6)	72 (43.4)	60 (35.3)	34 (21.5)	< 0.001∗∗∗
SDSS-collective activities (1–2)	150 (70.8)	112 (59.3)	77 (45.3)	47 (29.7)	< 0.001∗∗∗
SDSS-family activities (1–2)	67 (31.6)	72 (38.1)	50 (29.4)	25 (11.8)	< 0.001∗∗∗
SDSS-family function (1–2)	55 (25.9)	31 (16.4)	19 (11.2)	10 (6.3)	< 0.001∗∗∗
SDSS-self-care of personal life (1–2)	28 (13.2)	8 (4.2)	6 (3.5)	3 (1.9)	< 0.001∗∗∗
SDSS-external care and interest (1–2)	74 (34.9)	41 (21.7)	25 (14.7)	18 (11.4)	< 0.001∗∗∗
SDSS-sense of responsibility and planning (1–2)	76 (35.8)	43 (22.8)	36 (21.2)	9 (5.7)	< 0.001∗∗∗

∗∗∗*P* < 0.001; *P* value is the result of Cochran Armitage trend test.

a Patients in retirement/unemployed/sick leave status will not be evaluated; T0: 112; T1: 133; T2: 118; T3: 85.

b Patients in divorced/widowed/unmarried status will not be evaluated; T0: 32; T1: 25; T2: 12; T3: 10.

c Patients without children will not be evaluated; T0: 15; T1: 14; T2: 8; T3: 8.SDSS, Social Function Deficiency Screening Form.

#### Dimensions of SDSS

As shown in Table 3, the incidence of functional defects in the social withdrawal dimension and the collective activity dimension at four-time points remained high and ranked in the top three dimensions. The top three dimensions of functional defect incidence also included sense of responsibility and planning dimension, family activities dimension, and work function dimension. However, the dimensions of self-care in personal life, parental function, family function, and marriage function performed better. The Cochran-Armitage trend test results indicated a linear trend between time and the incidence of functional defects in each dimension (*P* ​< ​0.001).

### AOD

The total scores of Adaptation of Disability Scale Revised at T0, T1, T2, and T3 were 88.70 points (SD ​= ​12.43), 89.17 points (SD ​= ​15.45), 91.22 points (SD ​= ​14.50), and 92.91 points (SD ​= ​12.64), respectively, indicating an increase with time (*P* ​< ​0.001). However, the results of pairwise comparison at two adjacent time points indicated no statistical difference except between T2 and T3. Among the four dimensions, except for the difference in the E-dimension at different stages (*P* ​= ​0.432), the C-dimension (*P* ​= ​0.003), the T-dimension (*P* ​= ​0.015), and the S-dimension (*P* < 0.001), all showed statistically significant. These data are shown in Table 2.

### Factors influencing social participation

In this study, the total SDSS score was used as the response variable, and the demographic variables, disease- and treatment-associated variables, and AOD were separately used as factors or covariates to fit the linear GEE in analysis of the effect on social participation. Univariate analysis indicated significant differences in age, work status during diagnosis, residence, education, per capita monthly income of the family, surgery type, axillary lymph node dissection, postoperative chemot-herapy, radiotherapy, whether to discharge with portable drainage device, neoadjuvant chemotherapy, and AOD. These factors did not show multiple collinearity and were further included in the multivariate GEE of multiple factors. AOD (*P* ​< ​0.001), age (*P* ​= ​0.044), residence (*P* ​= ​0.007), surgery type (*P* ​= ​0.043), and postoperative chemotherapy (*P* ​= ​0.003) had statistically significant effects on social participation and were the key factors influencing social participation. These data are shown in Table 4.

**Table 4:** Multivariate analysis of factors influencing social participation.

Variable	*β*	*SE*	95% CI	Wald $\chi^{2}$	*P*
ADS	−0.09	0.01	−0.10∼−0.07	121.74	< 0.001∗∗∗
Age	0.03	0.01	<0.01–0.05	4.07	0.044∗
Residence				13.95	0.007∗∗
Countryside	0.96	0.39	0.20–1.73	6.12	0.013∗
Township	0.55	0.44	−0.31∼1.41	1.59	0.208
County town	1.02	0.30	0.43–1.60	11.56	< 0.001∗∗∗
Prefecture level city	0.68	0.26	0.17–1.18	6.91	0.009∗∗
Provincial capital	0^a^				
Surgery type				5.98	0.043∗
Total mastectomy	0.85	0.35	0.16–1.54	5.90	0.015∗
Modified radical mastectomy	0.37	0.31	−0.24∼0.99	1.41	0.236
Breast conserving surgery	0^a^				
Postoperative chemotherapy				8.99	0.003∗∗
Yes	0.59	0.20	0.99–0.21	8.99	0.003∗∗
No	0^a^				
Work status during diagnosis				0.23	0.631
On job	−0.12	0.25	−0.62∼0.37	0.23	0.631
Unemployed or retired	0^a^				
Axillary lymph node dissection				0.57	0.449
Yes	0.22	0.29	−0.35∼0.79	0.57	0.449
No	0^a^				
Radiotherapy				2.18	0.140
Yes	0.37	0.25	−0.12∼0.86	2.18	0.140
No	0^a^				
Education level				2.24	0.524
Bachelor degree or above	−0.41	0.29	−0.97∼0.15	2.07	0.150
Junior college	−0.29	0.33	−0.94∼0.36	0.77	0.382
High school or technical secondary school	−0.36	0.3	−0.97∼0.25	1.35	0.245
Junior high school or below	0^a^				
Family monthly income per capita (¥)				1.26	0.532
≥ 6000	−0.13	0.31	−0.74∼0.48	0.17	0.683
3001–6000	0.15	0.28	−0.39∼0.69	0.29	0.592
< 3000	0^a^				
Discharge with portable drainage device				1.25	0.264
Yes	−0.33	0.29	−0.90∼0.25	1.25	0.264
No	0^a^				
Diagnosis type				<0.01	0.962
Invasive carcinoma	−0.01	0.25	−0.50∼0.48	<0.01	0.962
Carcinoma *in situ*	0^a^				
Neoadjuvant chemotherapy				0.91	0.341
Yes	0.27	0.28	−0.28∼0.82	0.91	0.341
No	0^a^				

0^a^, reference group; β, Partial regression coefficient; SE, standard error; Wald, Wald statistics.

∗*P* < 0.05; ∗∗*P* < 0.01; ∗∗∗*P* < 0.001.

To explore the dynamic effects of AOD on social participation, we considered the total SDSS score as the response variable, and time and AOD as factors; included only the interaction of time and AOD in the model; used GEE to analyze the effects of interaction on social participation. The results demonstrated a statistical difference (*P* ​< ​0.001), thereby indicating that the influence of AOD on social participation varied at different times. These data are shown in Table 5.

**Table 5:** The dynamic impact of AOD on social participation.

Variable	*β*	*SE*	95% CI	Wald $\chi^{2}$	*P*
T∗ ADS				291.59	< 0.001∗∗∗
T0∗ ADS	−0.09	0.01	−0.10∼120.73	137.83	< 0.001∗∗∗
T1∗ ADS	−0.10	0.01	−0.11∼−0.08	167.45	< 0.001∗∗∗
T2∗ ADS	−0.10	0.01	−0.12∼−0.09	193.59	< 0.001∗∗∗
T3∗ ADS	−0.11	0.01	−0.12∼−0.09	214.08	< 0.001∗∗∗

β, Partial regression coefficient; SE, standard error; Wald, Wald statistics; ∗∗∗*P* < 0.001.

## Discussion

Although differences in demographic data were present between the follow-up group and the lost group at baseline, no significant difference was observed in the levels of social participation and AOD between groups. Therefore, the difference had little effect on the comparability of outcomes.

### Dynamic trend in social participation

Six months after surgery was a critical period for patients with breast cancer to re-adapt to society. During this period, the social participation of patients with breast cancer improved over time, consistent with previous studies.^16^^,^^17^^,^^26^ The reasons underlying this improvement might have been that patients might receive substantial care from their families in the short-term after surgery. Patients’ physical functions gradually recover over time, and they could gradually assume family responsibilities, return to work, and plan for the future. Another explanation might have been that the patients gradually accepted the disease and the changes in their body image after surgery, and began to try activities outside the family, thus increasing their social participation.

However, the overall social participation status was poor, particularly in social dimensions including social withdrawal, collective activities, sense of responsibility and planning, and work function, whereas family dimensions including marriage function, parental function, and self-care ability were relatively good. Therefore, patients had greater difficulty in maintaining their social activities than in their everyday activities. Medical staff should inform patients of the importance of returning to society to aid in their physical and mental recovery.^26^ They should establish contact with patients and follow up for at least 6 months, regularly ask them if they have difficulties in social participation, formulate effective interventions to help them cope and adapt effectively, encourage them to rebuild their social circle and improve social participation.

### Dynamic trend in AOD

The AOD among young and middle-aged patients having breast cancer within 6 months after surgery was moderate, and their disability acceptance was improved over time, which was consistent with previous studies.^18^^,^^19^ The patient's self-care ability continually improved over time, the treatment effect had shown, and the “patient role” had been weakened. In addition, patients gradually accepted and adapted to the disease. They have shifted from focusing on disease- and treatment-associated issues to focusing on improving their own value, and their AOD increased.

### Dynamic influence of AOD on social participation

AOD was the main factor influencing social participation, and the effect of AOD on social participation increased over time. The dysfunction and appearance changes caused by surgery and treatment profoundly affect patients with breast cancer. These changes continually remind patients of their “cancer patient identity”, thus making them feel less sexually attractive and less feminine and diminishing their sense of self identity, thus affecting their social participation. When encountering negative life events, women were more inclined to think about negative effects repeatedly. The accumulation of negative effects might, therefore, have a stronger hindering effect on social participation. Medical staff should pay attention to the evaluation of AOD in patients with breast cancer after surgery, focus on patients with low AOD, develop practical interventions to improve AOD, and further promote social participation.

### Critical factors influencing social participation

Age, residence, surgery type, and postoperative chemotherapy were the main factors influencing social participation. Among the young and middle-aged patients, older age patients were associated with slower recovery of physical function, more serious adverse effects caused by treatment, and the more serious social function defects. Most of these patients were retired or had retired early due to illness and had few social activities and poor adaptability, thus hindering their integration into social interaction. However, the young patients were in rising stages of their career and had a strong sense of family responsibility. The economic burden caused by the treatment might, therefore, have prompted them to adjust their mentality and return to work as soon as possible. Medical staff should focus on older young and middle-aged patients, inform them of the importance of social integration, and encourage them to participate in social interaction.

Patients living in rural areas, county towns, or prefecture-level cities had a poorer level of social participation than those living in provincial capital cities, possibly because patients living in provincial capital cities had convenient medical treatment, high treatment compliance, and stronger confidence in overcoming diseases. Moreover, they often had more access to social resources, more job opportunities, and more interpersonal and social interaction, thus facilitating better social participation.^27^

Breast conserving surgery has been found to be a factor promoting social participation.^28^^,^^29^ Patients who received breast conserving surgery tended to have mild conditions with small tumors. Because most of them needed radiotherapy but not chemotherapy after surgery, the psychological burden of patients was relatively light. Whereas the loss of breast had a strong psychological effect on patients, breast conserving surgery could well maintain the original shape of the breast, thus resulting in fewer changes in patients’ shape and less impact on social participation. Medical staff should focus on patients receiving total mastectomy or modified radical mastectomy and provide targeted appearance care plans to help them cope with body changes, such as wearing artificial breast and breast reconstruction, so as to overcome social barriers.^30^

Social participation was relatively poorer among patients receiving chemotherapy after surgery, in agreement with previous research results.^28^ The adverse effects of chemotherapy, such as nausea, vomiting, anorexia, and fatigue, were substantial. In addition, chemotherapy often led to clear appearance changes, such as hair loss, eyebrow loss, pigmentation, edema, and so on, which might potentially lead to body image problems, and cause them to fear others’ gaze and avoid social interaction. Medical staff should encourage patients to communicate with their wardmates about experiences in coping with adverse effects, reduce social phobia, and help them better recover and return to society.

### Strengths and limitations

To our knowledge, this was the first longitudinal study to explore the changes in social participation and AOD of patients with breast cancer after surgery. Our finding confirmed that the negative effect of AOD on social participation accumulated over time. However, we acknowledged several study limitations: (1) the survey site was limited to one hospital and the representativeness of the results required further consideration; (2) the limited sample size might cause some potential influencing factors not to show statistical differences; (3) the follow-up time was relatively short and the number of follow-up was small. Future studies must expand the research scope, increase the sample size, extend the follow-up time, increase the number of follow-up visits, and further explore the dynamic trend in social participation and AOD, as well as the possible effect of other related factors on social participation.

## Conclusions

The social participation and AOD among young and middle-aged patients with breast cancer after surgery showed a trend of dynamic improvement, but social participation remained poor. Medical staff should establish contact with young and middle-aged patients with breast cancer, focusing on older patients who live outside the provincial capital city, and who receive total mastectomy or modified radical mastectomy and postoperative chemotherapy. AOD might be an important potential avenue for improving the level of social participation level of breast cancer patients after surgery.

## Data Availability

The datasets generated during and analyzed during the current study are available from the corresponding author on reasonable request.

## CRediT author statement

**Mengyao Zhu**: Conceptualization, Methodology, Investigation, Software, Data curation, Writing - Original draft. **Yiheng Zhang**: Conceptualization, Methodology and Investigation. **Haiyan He, Lili Chen and Juanjuan Chen**: Investigation and Supervision. **Meifen Zhang:** Writing- Reviewing, Editing and Supervision.

## References

[1] World Health Organization (2020). https://www.iarc.fr/faq/latest-global-cancer-data-2020-qa.

[2] Mc Donough M.H., Sabiston C.M., Wrosch C. (2014). Predicting changes in posttraumatic growth and subjective well-being among breast cancer survivors: the role of social support and stress. Psycho Oncol.

[3] World Health Organization (2001).

[4] Lv L.M., Zhang X.H., Wang X.X., Zhang J.L. (2017). A study on subthreshold depression and social participation among young and middle-aged patients after breast cancer surgery. J Nurs Sci.

[5] Cao G. (2018). Correlation between depression and social dysfunction in middle-aged patients with breast cancer after operation. Chinese Rural Health Service Administration.

[6] Thraen-Borowski K.M., Trentham-Dietz A., Edwards D.F., Koltyn K.F., Colbert L.H. (2013). Dose-response relationships between physical activity, social participation, and health-related quality of life in colorectal cancer survivors. J Cancer Surviv.

[7] Kaya C., Chan F., Tansey T. (2019). Evaluating the world health organization's international classification of functioning, disability and health framework as a participation model for cancer survivors in Turkey. Rehabil Counsel Bull.

[8] Beesley V.L., Vallance J.K., Mihala G., Lynch B.M., Gordon L.G. (2017). Association between change in employment participation and quality of life in middleaged colorectal cancer survivors compared with general population controls. Psycho Oncol.

[9] Yang Z.Y., Chen W.L., Wu W.T., Lai C.H., Ho C.L., Wang C.C. (2022). Return to work and mortality in breast cancer survivors: a 11-year longitudinal study. Int J Environ Res Publ Health.

[10] Su T.T., Azzani M., Tan F.L., Loh S.Y. (2018). Breast cancer survivors: return to work and wage loss in selected hospitals in Malaysia. Support Care Cancer.

[11] Sun F.K., Lu C.Y., Yao Y., Chiang C.Y. (2023). Social functioning, depression, and quality of life among breast cancer patients: a path analysis. Eur J Oncol Nurs.

[12] Grayson M. (1951). Concept of “acceptance” in physical rehabilitation. J Am Med Assoc.

[13] Tuenchai A.T., Apichana K.O. (2005). The infuencing factors of acceptance of disability in spinal cord injured patients. Nepal J Neurisci.

[14] Li L., Wu X.L., Xu L. (2018). Factors affecting self-perceived participation and autonomy among patients with burns: a follow-up study. Burns.

[15] Zhang Q.Y., Xiao S., Yan L. (2019). Psychosocial predictors of adjustment to disability among patients with breast cancer: a cross-sectional descriptive study. J Nurs Res.

[16] Fallahpour M., Jonsson H., Joghataei M.T., Kottorp A. (2011). Impact on Participation and Autonomy (IPA): psychometric evaluation of the Persian version to use for persons with stroke. Scand J Occup Ther.

[17] Tse T., Linden T., Churilov L. (2019). Longitudinal changes in activity participation in the first year post-stroke and association with depressive symptoms. Disabil Rehabil.

[18] Chiu S.Y., Livneh H., Tsao L.L., Tsai T.Y. (2013). Acceptance of disability and its predictors among stroke patients in Taiwan. BMC Neurol.

[19] Chen R., Kotbungkair W., Becton A. (2015). A comparison of self-acceptance of disability between Thai buddhists and American christians. J Rehabil.

[20] Li L.L., Li X.M., Han D.F. (2020). A longitudinal study of identification and predication of psychological distress trajectories among breast cancer patients. Chin J Nurs.

[21] Zhuo D.H. (1990).

[22] Liu F.L. (2008). Society participation state of breast cancer patients and its influencing factors. Chin Nurs Res.

[23] Groomes D., Linkowski D.C. (2007). Examining the structure of the revised acceptance disability scale. J Rehabil.

[24] Chen N., Chen Y., Sun X.C., Hu J.J. (2009). Relationship between acceptance of disability and posttraumatic stress response in patientswith brachial plexus injury. Chin J Nurs.

[25] Dorland H.F., Abma F.I., Roelen C.A.M. (2018). Work-specific cognitive symptoms and the role of work characteristics, fatigue, and depressive symptoms in cancer patients during 18 months post return to work. Psycho Oncol.

[26] Nikolić S., Ilić-Stosović D., Kolarević I. (2015). Social participation of women with breast cancer. Vojnosanit Pregl.

[27] Yang S.S., Liu J.E., Su Y.L., Zhao Y., Liu Y.F. (2020). The investigation on the status and influence factors of returning to work among breast cancer survivors. Chinese Nursing Management.

[28] Hou S.Q., Lu Z.Q., Qiu J.J. (2015). Social avoidance and distress in breast cancer patients and the associated factors. Journal of Nursing Science.

[29] Zhou Y.C. (2021). Effects of different surgical methods on social function and quality of life of patients with breast cancer. Maternal and Child Health Care of China.

[30] Zhu M., Sun S., Zhang Y. (2022). Effects of the appearance care on psychosocial outcomes for breast cancer: a systematic review and meta-analysis. Support Care Cancer.

